# Clinical Case Reports on the acceptability and tolerance of a High‐Energy whey peptide‐based Pediatric oral nutritional supplement in children aged over 12 months

**DOI:** 10.1002/ccr3.4887

**Published:** 2021-10-05

**Authors:** Kathryn Simpson

**Affiliations:** ^1^ CCICP – Central Cheshire Integrated Care Partnership Crewe UK; ^2^ Mid Cheshire Hospital's NHS Trust Crewe UK

**Keywords:** acceptability, gastrointestinal tolerance, nutrition, ONS (oral nutritional supplements), pediatric

## Abstract

The nutritional management of the complex needs of children with impaired gastrointestinal function can be challenging, using a high‐energy pediatric whey‐based peptide formula in clinical practice demonstrates its role in managing symptoms.

## Graphical abstract

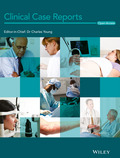

## 1 INTRODUCTION

Oral nutritional supplements may reduce the risk and impact of disease‐related malnutrition on children, however, compliance may be an issue in those with GI dysfunction, these case reports demonstrate the potential role of whey peptide‐based formulae with a range of clinical conditions either orally or via a feeding tube.

The management of feeding intolerances in children with complex medical conditions is very individualized and requires extensive dietetic support. These clinical case reports demonstrate the use of a high energy pediatric whey peptide formula in clinical practice.

Multiple clinical conditions can lead to gastrointestinal disorders and intolerance to feeding that dramatically affect the nutrition and health status of patients. 75% of critically ill children and up to 90% of chronically ill children may be malnourished or have insufficient nutrition.^1^, ^2^, ^3^, ^4^, ^5^, ^6^, ^7^, ^8^, ^9^, ^10^ Malnutrition in children leads to impaired growth and development and worse clinical outcomes.^11^, ^12^

Critically and chronically ill children who require nutritional support and are intolerant to polymeric feeds may be eligible to use a semi‐elemental formula in an oral format, oral feeding being the favored feeding route, where possible.^13^, ^14^, ^15^, ^16^, ^17^, ^18^ Indeed, oral feeding in children is important to prevent oral hypersensitivity and feed aversion and promote the development of oral‐motor feeding skills.^17^

Oral nutritional supplements (ONS) may reduce the risk of malnutrition in children. More specifically, whey peptide‐based formulas may reduce the frequency of reflux, GORD and allowing better digestion and absorption of nutrients, especially for those children with complex gastrointestinal problems.

Orally feeding a child with chronic illness may lead to excessive amounts of time each day spent on feeding, severely impairing parent and caregiver QoL.^19^ A retrospective chart review saw improved feeding tolerance in 92% of patients within one week of switching to a peptide‐based feed (Peptamen Junior) in 13 children with developmental delay who failed to reach their nutritional goals using standard polymeric formulas.^20^

These clinical cases illustrate the dietetic management of children with conditions that pose a risk of malnutrition. Providing nutrition in the form of peptides can support a child's growth, meet nutritional requirements, and support better a quality of life.

Enteral feeding is the preferred method of providing nutritional support to children who have a functioning gastrointestinal tract; some children receive their full nutritional requirements via enteral tubes, whereas others require nutritional support to supplement poor oral intake or meet increased nutritional requirements. Enteral feeding can be short‐term, or a life‐long method of feeding, with feeding regimes adapted for each child to meet individual nutritional requirements.^21^ In the UK, indication for enteral nutritional support in children is supported by the PENG (parental and enteral nutrition group) detailing indication for enteral nutritional support, types of feeding, indications, and contraindications.^22^

## 2 CASE STUDY 1. THE USE OF A PEDIATRIC PEPTIDE FORMULA FOR A RARE CONGENITAL BIRTH DEFECTS CHILD

### 2.1 Summary

Tracheoesophageal fistula (TOF) and esophageal atresia (OA) are rare congenital birth defects seen in approximately 1 in 3500 births.^23^ H is a 6‐year‐old girl who had been under dietetic review since being discharged home as an infant, medical support continues from the tertiary TOF multidisciplinary team and local services. GORD and loose stools remained problematic. H had no worsening symptoms regarding her GORD or bowel frequency when taking the Peptamen Junior 1.5 orally or given via feeding tube. H was reported to like only the banana‐flavored ONS and preferred to drink it via a straw.

### 2.2 Clinical case study description

Tracheoesophageal fistula (TOF) and esophageal atresia (OA) are rare congenital birth defects seen in approximately 1 in 3500 births.^23^ Surgery is required within the first few days of life. Despite excellent survival rates, infants are at risk of developing several morbidities, including gastro‐esophageal reflux (GORD), esophageal stricture, feeding difficulties, and respiratory problems. These difficulties are more problematic during the first year of life; however, problems can persist for life.

As many as 50% of infants with TOF and OA also have other congenital defects, the most common being VACTERL complex affecting multiple organ structures,^24^ residual problems may cause long‐term disability, the severity of which will vary depending on the number and type of defects. Up to 68% of patients with TOF experienced feeding difficulties, including pain, regurgitation, and vomiting.^25^ For many children with complex TOF and OA, enteral tube feeding is necessary as feeding problems persist, these usually improve as the child grows older and they develop their own coping strategies, for some growth is an issue due to failure to thrive and scoliosis.

For some OA babies, the distance between the upper and lower ends of the esophagus are impossible to join – classified as long gap OA, up to half of these children can experience feeding difficulties still at 7 years of age.^26^ Children often show difficulty in feeding and a reluctance to swallow; strictures require repeated dilatations and experience repeated choking and coughing mealtimes become stressful and a very slow process. Many children who have undergone gastric tube interposition their weight and height fell below the 10^th^ percentile.^27^

### 2.3 Medical history

H is a 6‐year‐old girl who has been under dietetic review since being discharged home as an infant, medical support continues from the tertiary TOF multidisciplinary team and local services.

H was born at 36 weeks gestation weighing 2.44 kg (9th centile); she presented with TOF, OA, VACTERL association. H required a two‐stage surgical procedure due to her presenting condition's complexity (long gap OA). Initial surgery included a thoracotomy, gastrostomy, esophagostomy, and ligation, and then a further thoracotomy aged 3 weeks and later corrective surgery – gastric tube esophagoplasty, at the age of 2 when a Jejunostomy tube was cited, long‐term iron supplementation was commenced. H was orally averse despite sham feeding initially with infant formula and then weaning foods to allow normal development and coordination. GORD and loose stools remained problematic despite changes in infant formula and medical management, a peptide formula was trialed, and symptom improvement was evident.

H at the age of 4 required repeated esophageal dilatations every 4–6 weeks. This led to heightened food aversion and poor oral feeding; this was in line with her complex anatomy and previous surgical interventions. The cycle of esophageal stretching meant a slower process of accepting foods due to slowed oral‐motor development.

H started school and began to make gradual progress with her oral skills supported by the speech therapy and dietitian working in collaboration to amend her enteral tube feeds, allowing different food textures, tastes, consistencies to be offered, this helped to speed up the process of finding what she could cope with and what food caused her to struggle, often it was one step forward and two steps back with oral feeding.

H at the age of 5 began the gradual reduction of her Jejunostomy feeds as oral skills were continuing to improve. Feeds were gradually reduced from 900 ml Nutrini peptisorb energy (Nutricia) to 700 ml given from 4 pm throughout the night, allowing oral diet to be encouraged further during the day while still maintaining weight gain.

H oral skills continued to improve but food quantity remained an on‐going issue, and any further reduction in enteral feeds led to weight loss. H’s development had progressed well, she was now able to communicate her wishes and feeling and identifying the foods she could manage. H had previously tried a peptide ONS, Paediasure peptide (Abbott Nutrition) but disliked the taste and refused to drink it; all other polymeric pediatric ONS were not tolerated.

The introduction of Peptamen Junior 1.5 (Nestle Health Science) oral nutritional supplement (ONS) allowed H to reduce enteral tube feeding volumes to 700 ml total feed, 500 ml overnight, and 200 ml either as a ONS or as an enteral tube feed top‐up. The initial plan was to offer 100 ml peptamen junior‐1.5 mid‐morning and 100 ml mid‐afternoon via a cup and straw. H would only drink the Banana‐flavored ONS and most days achieved the 2 × 100 ml orally – the introduction of the ONS made no difference to her GORD or bowel movements.

### 2.4 Nutritional intervention

The nutritional aim was to meet her nutritional requirements of 1545 kcal and 28.3 g protein per day from a combination of oral diet, oral ONS, overnight jejunostomy feeding.

The objectives were to reduce the reliance on enteral tube feeding, maximize feed absorption, and reduce GORD symptoms if possible while ensuring age‐appropriate weight gain and growth.

Weight was recorded at 16.8 kg (2nd centile), Height – 98 cm (<0.4th centile) BMI – 17.5 (75–91st centile).

### 2.5 Outcome

H was reviewed via telephone after 2 days to establish whether she was managing to take the ONS orally and at home after a full 7‐day trial. H was reported to only drink the banana‐flavored ONS via a straw. Over the 7‐day trial, 4 top‐up feeds from a possible 14 needed to be given via her Jejunostomy tube to ensure the full daily volume for 200 ml. Feeds given via jejunostomy ranged from 35 to 100 ml. H asked for the drink without prompting and remained very positive that she could continue drinking it as she liked the smell, taste, and texture of the drink. Mum commented that this was the best she had ever seen her drink.

H had no worsening symptoms regarding her GORD or bowel frequency when taken orally or given via feeding tube. H commented that she wanted to continue with the drink as “she then didn't need to be on her pump feed as long, she could play more after tea”.

### 2.6 Discussion

H had exceeded expectations in taking the ONS orally and only needing occasional top up's via her feeding tube; she liked the taste and was happy to drink it twice a day. The ONS also provided a comparable peptide nutritional alternative to her overnight feed and was well tolerated. H’s weight remained stable during the trial, the family expressed their wish to continue with the ONS at the end of the trial as her quality of life was improving, as she was less reliant on the overnight feed, the family were reassured that she was still meeting her nutritional requirements.

The use of a high calorie pediatric peptide ONS for H allowed flexibility within her feeding plan and maintained an appropriate nutritional intake allowing H to only commence overnight feeds when she went to bed rather than early evening allowing her more time at her evening meal to eat slowly and improve her chewing skills with the support of her family and without the pressure of time to commence her overnight enteral feed.

### 2.7 Conclusion

Feeding problems in children with complex TOF, OA, VACTERL association can be very challenging, adequate nutritional support can be difficult to achieve in the older child who is trying to normalize life and cope with the associated problems.

The introduction of a high calorie peptide ONS which is well tolerated and can be used as an oral supplement or bolus feed in children with complex medical needs can only enhance a child's nutritional options, increase convenience, improve oral feeding, and support a better quality of life.

## 3 CASE STUDY 2: COCKAYNE SYNDROME AND TOLERANCE TOWARD A HIGHER ENERGY PEPTIDE FORMULA

### 3.1 Summary

Cockayne syndrome is a rare disorder characterized by short stature and an appearance of premature aging.^28^ S is an 8‐year‐old girl who has been under dietetic review since she was 32 weeks old, due to concerns around her nutritional intake, reflux, and poor tolerance of infant polymeric formula. All attempts at calorie additions to her feeding plan resulted in increased reflux, vomiting, and reduced feed tolerance despite using medications to suppress these tolerance issues. Modular supplements increased feed volume, amendments to feeding rates and regimens always resulted in increased intolerance symptoms, thus limiting any effective increase in daily calories and weight gain. For this child, a change in feed to Peptamen Junior 1.5 (Nestle Health Science) ONS increased her daily enteral calories providing ¾ of her daily requirements. The introduction of the high calorie peptide feed allowed her usual daily routine to be followed with an increased nutrient intake.

### 3.2 Clinical case study description

Cockayne syndrome occurs in about 2.7 per million live births in Western Europe.^28^ It can be divided into subtypes which are distinguished by the severity and age of onset of symptoms. Type 1 is the classical form characterized by normal fetal growth with onset of abnormalities in the first two years of life as seen in this case report.

There is no cure and treatments are based around specific symptoms as they appear, no evidence currently exists on the role of nutrition in supporting this condition. Feeding and nutritional status are significant concerns in Cockayne syndrome, the growth of children with this syndrome often falls across centiles before a diagnosis is made.^28^, ^29^

Features of Cockayne syndrome include a failure to gain weight and growth at the expected rate (failure to thrive), abnormally small head size (microcephaly), and impaired development of the nervous system.^28^, ^29^ Malnutrition in children leads to impaired growth and development and worse clinical outcomes.^30^ In children with neurological impairment and compromised GI function who require EN with a semi‐elemental formula (N = 8), switching from another semi‐elemental formula to a Peptamen Junior formula containing 1.5 kcal/ml* for 21 days resulted in increased calorie intake, such that patients met their calorie intake goals.^31^

Cockayne syndrome is a rare disorder characterized by short stature and an appearance of premature aging. Features of this disorder include a failure to gain weight and grow at the expected rate (failure to thrive), abnormally small head size (microcephaly), and impaired development of the nervous system. Other common features include photosensitivity, hearing loss, eye abnormalities, severe tooth decay, bone abnormalities, and brain changes.^32^

### 3.3 Medical history

S is now an 8‐year‐old girl who has been under dietetic review since she was 32 weeks old; she was initially referred due to concerns around her nutritional intake, reflux, and poor tolerance of infant polymeric formula. She was born at term with a birth weight of 2.76 kg (2nd−9th centile) with no other neonatal problems. S was commenced on Pepti‐junior (Nutricia) along with ranitidine and domperidone for the reflux.

By 1 year of age, S anthropometrics ‐ weight 6.22 kg (<0.4th centile), height 66.1 cm (<0.4th centile) she was only managing minimal amounts of Pepti‐Junior via bottle and a small amount of milk‐free weaning foods. At this point, her feeds were swapped to Infatrini peptisorb (Nutricia) to try to maximize her calorie intake without increasing milk volume due to tolerance issues.

By the time she was 18‐months‐old, she had been referred to Metabolic and Genetics team for further investigations. S began needing more support with her day‐to‐day functioning, she was admitted for feeding observation and initiation of supplementary NG tube feeding. Speech therapy input highlighted that S oral feeding skills were developing, but she displayed aversive feeding behaviors toward eating and drinking and did not appear to show any signs of hunger. The range and quantity of her food were extremely limited, reliance on supplementary tube feeding was increasing. A feed thickener was added due to aspiration concerns was expressed about aspirating when drinking; a videofluoroscopy (VF) was requested.

At 2 years of age, global development delay was highlighted, S was known to a wide range of professionals to support her increasing needs, a PEG was inserted, and this was replaced by a button device 6 months later.

At the age of 3 years, S was diagnosed with Cockayne syndrome Type 1, her weight and height continued to track just below the 0.4th centile. Enteral feeds were swopped to an amino acid formula Neocate (Nutricia) to try to maximize tolerance, and she continued to be disinterested in food and oral fluids.

At 5½ years, she was reported to be developmental stage 22–36‐month stage and receiving support from a wide range of services, she remained mainly gastrostomy fed and growing below the 0.4th centile. Gastrostomy feeds continued with an Amino acid formula; this was swapped to Neocate Junior (Nutricia), aiming to provide additional calories to promote weight while maintaining restricted volumes tolerated without causing increased reflux, retching, vomiting. This was swapped after 3 months to Paediasure peptide (Abbott Nutrition) due to its ease in use (readymade formula) and easier transport to school, and respite care and poor tolerance of the higher calorie feed. Discussions also took place regarding alternative enteral routes, but parents expressed a wish to continue with PEG feeding.

S weight continued below the 0.4th centile, all attempts at calorie additions to her feeding plan resulted in increased reflux, vomiting, reduced feed tolerance despite the use of medications to suppress these tolerance issues. The use of both modular supplements increased feed volume and amendments to feeding rates and regimens always resulted in increased intolerance symptoms, thus limiting any effective increase in daily calories and weight gain. With the support of school and siblings at home, S began eating more food, the quantities remained small and food variety was limited. Feed tolerance was always reduced further when s was unwell. S had also reviewed at the specialist rare diseases clinic, who again suggested using modular supplementation, but again symptoms of reflux and GI intolerance were experienced.

S continued to vomit despite the changes in formulas, amended feed times – daytime versus overnight feeding, and combination feeding and trialing with the different feeding options of a continuous pump, pump‐assisted bolus and bolus feeding methods. The clinical decision made was that the current pump‐assisted feeding was deemed the safest option and suited family lifestyle.

S feeding plan consisted of 4 pump‐assisted bolus feeds of Paediasure Peptide, providing 640 kcal and 18 g protein/day excluding oral diet, therefore approximately half of her nutritional requirements.

The introduction of Peptamen Junior 1.5 (Nestle Health Science) bottles allowed S to continue with bolus feeds at the same volume for each feed as the previous 1.0 kcal/ml feed, feeds were reduced on the day of introduction from 160ml to 120mls to assess tolerance, as there were no symptoms evident parents decided on Day 2 to revert back to the 160mls, they continued at the volume for the trial duration.

### 3.4 Nutritional intervention

The nutritional aim was to meet her nutritional requirements of 1230 kcal and 14.5 g protein per day (corrected for weight age) from the oral diet as tolerated and her 4 bolus PEG feeds to allow S to continue to mobilize and access food at mealtimes.

The objectives were to continue with pump‐assisted bolus feeding with either a reduced feed volume fed to allow more time away from feeds without reducing the overall calorie intake or, if tolerated well, to increase the overall calories provided from the enteral feed.

Weight was recorded at 11.95 kg (<<0.4th centile), Height – 101.5 cm (<<0.4th centile) BMI −11.6 (<0.4th centile).

### 3.5 Outcome

S was reviewed via telephone after 2 days to establish ONS tolerance; feeds were set at the normal rate and volume. S was reviewed again at the end of the 7‐day trial; she had tried it via a cup but refused to drink it; therefore, all feeds were given as expected via her gastrostomy tube, she achieved the full volume of feed daily along with additional water flushes for hydration.

This change in feed increased her enteral nutrition to 960 kcal and 29 g protein/day, therefore providing ¾ of her daily requirements excluding oral intake. As S was able to tolerate her usual feed volume without affecting symptoms and no oral intake reduction, weight gain should be seen with longer‐term use.

### 3.6 Discussion

S had exceeded expectations in tolerating the Peptamen Junior bottles at the same rate and volume as her usual lower calorie feed, no worsening symptoms were experienced, in view of the improved nutritional profile, the family expressed their wish to continue with the enteral feed as they felt she was gaining weight and finally able to tolerate more calories than she had ever done before. Food intake remained stable, S continued to enjoy small meals with her family.

The introduction of the high calorie peptide feed allowed her usual daily routine to be followed while also increasing nutrient intake.

### 3.7 Conclusion

S was able to tolerate the same volume of feed as the previous lower calorie feed, therefore improving her overall nutritional intake. This provided reassurance to parents that nutritional intake was optimized and allowed S to maintain quality of life by avoiding prolonged feeding times. The milk was well tolerated and may be related to the high MCT content, which is more readily absorbed.

The use of a high calorie MCT dominant ONS and medical management may help to reduce the feeding and nutritional concerns that remain significant concerns in Cockayne syndrome.

## 4 OVERALL CONCLUSION

These case studies illustrate the difficulties in managing feeding intolerances often associated with rare conditions such as congenital disease and Cockayne syndrome. These children tend to exhaust different formula options before responding to the benefits of a peptide formula. Early consideration of a high energy peptide formula may reduce the unpleasant experience of reflux or other GI symptoms and help promote normal growth among these children.

## AUTHOR CONTRIBUTIONS

Informed consent was obtained from the patient's guardian's.

## ETHICAL APPROVAL

These clinical case reports involving children over 12 months of age are presented in accordance with the ethical standards of the Health Research Authority and Mid Cheshire Hospital's NHS Foundation Trust policy framework for Health and Social care research.

## CONSENT

The author gained verbal and written consent for publication from these two patients, consent was obtained from the parent with parental responsibility of each child.

## Data Availability

Data available on request from the author due to privacy/ethical restrictions.
